# Phytobiotics from Oregano Extracts Enhance the Intestinal Health and Growth Performance of Pigs

**DOI:** 10.3390/antiox11102066

**Published:** 2022-10-20

**Authors:** Marcos Elias Duarte, Sung Woo Kim

**Affiliations:** Department of Animal Science, North Carolina State University, Raleigh, NC 27695, USA

**Keywords:** growth performance, oxidative stress, phytobiotics, pigs

## Abstract

This study aimed to investigate the effects of phytobiotics on the intestinal health and growth performance of pigs. Totals of 40 newly-weaned pigs with 6.4 ± 0.3 kg BW (Exp. 1) and 120 growing pigs with 27.9 ± 2.3 kg BW (Exp. 2) were allotted in RCBD in a 2 × 2 factorial arrangement. The factors were: antibiotics as growth promoter (AGP) and phytobiotics (PHY). Pigs were fed experimental diets during 21 d (Exp. 1) and 42 d (Exp. 2). Growth performance, health parameters, and nutrient digestibility were evaluated. In Exp. 1, AGP diet increased (*p* < 0.05) ADG and G:F compared with a diet without AGP or PHY and a diet with AGP combined with PHY. PHY decreased (*p* < 0.05) TNF-α and IgG in the jejunum and protein carbonyl in plasma, whereas it increased (*p* < 0.05) the villus height. In Exp. 2, AGP or PHY diets increased (*p* < 0.05) ADG, ADFI, and G:F compared with a diet without AGP or PHY and a diet with AGP combined with PHY. PHY decreased (*p* < 0.05) IgG and PC in plasma. Collectively, AGP and PHY improved growth performance by reducing oxidative stress and enhancing immune status and jejunal morphology. However, the combinational use of phytobiotics with antibiotics suppressed their effect.

## 1. Introduction

In swine production, antibiotics as growth promoter (AGP) have been used since the 1950s [1,2]. AGP can suppress or inhibit the proliferation of certain microorganisms, preventing or mitigating the severity of disease caused by microbial infection [1,3,4]. In addition, the reduction in pathogenic microbes would enhance intestinal health by reducing the inflammatory response and oxidative stress, ultimately improving the efficiency of nutrient utilization and promoting growth performance [2,5]. However, the residual antibiotic in animal products and the prolonged use of antibiotics in animal feeds can increase microbial resistance [6,7]. Due to public awareness regarding microbial resistance, the use of AGP in animal feeds has been banned or restricted around the world [4,6,7,8]. 

The restricted use of AGP has increased the challenges to the swine industry and researchers to maintain the productivity of pigs fed AGP-free diets. Numerous strategies have been attempted to overcome these challenges and produce healthy animals [9,10,11,12]. Among those alternatives, phytobiotics are bioactive compounds extracted from plants and algae with antimicrobial, anti-inflammatory, and antioxidant properties, which can be used in animal feeds to enhance health growth performance [13]. Moita et al. [14] reported that the use of a blend of plant-based functional oils can modulate the mucosa-associated microbiota and enhance intestinal health by reducing oxidative stress and enhancing jejunal morphology in nursery pigs. Moreover, Holanda et al. [11] reported that herbal extracts can mitigate the deleterious effects of mycotoxin challenges in nursery pigs due to the antioxidant properties of those compounds. The bioactive compounds in phytobiotics are generally related to phenolic compounds [13,15,16]. Extracts from oregano mainly contain carvacrol, thymol, γ-terpinene, and *p*-cymene, which have been reported to improve the health and performance of pigs through their antimicrobial and antioxidant properties [17,18,19].

Therefore, it was hypothesized that phytobiotics can be used as an alternative to antibiotics to promote the growth performance of pigs during nursery and growing phases by reducing the inflammatory response and oxidative stress in the small intestine and enhancing intestinal barrier function and nutrient utilization. Thus, this study aimed to investigate the effects of phytobiotics and antibiotics, singularly or in combination, on growth performance, intestinal immune status and oxidative stress status, intestinal barrier function, and nutrient digestibility in pigs during nursery and growing phases.

## 2. Materials and Methods

The Institutional Animal Care and Use Committee at North Carolina State University reviewed and approved the experimental procedures used in Exp. 1 and 2 in this study, following the North Carolina State Animal Care and Use Procedures (REG 10.10.01).

### 2.1. Animals, Experimental Design, and Diets

In Exp. 1, 40 newly weaned pigs (20 barrows and 20 gilts) at 21 d of age with initial body weight (BW) at 6.4 ± 0.3 kg were allotted based on a randomized complete block design, with sex (barrows and gilts) and initial BW (light and heavy) as blocks to four treatments based in a 2 × 2 factorial arrangement. Each treatment had 10 pens (*n* = 10; five pens with barrows and five pens with gilts), and the pigs were housed individually in pens (1.50 m × 0.74 m) with slatted floors. The first factor was the antibiotic as growth promoter (AGP; 0 or 390 mg chlortetracycline/kg diet + 33 mg tiamulin/kg of diet during phase 1 and 22 mg carbadox/kg of diet during phase 2; *n* = 20), and the second factor was the phytobiotics (PHY; 0 or 0.05%; *n* = 20). The experimental diets were formulated meeting the nutritional requirements suggested by NRC [20] and fed to pigs for 21 d based on 2 phases: phase 1 (d 1 to 7) and phase 2 (d 7 to 21). The composition of the basal diets is shown in Table 1. During the last 5 days of the experimental period, titanium dioxide (0.4%) was added to the diet as an indigestible marker to evaluate the nutrient digestibility [10]. 

In Exp. 2, 120 growing pigs (60 barrows and 60 gilts) at 27.9 ± 2.3 kg BW were allotted based on a randomized complete block design with sex (barrows and gilts) and initial BW (light and heavy) to four treatments. Each treatment had 10 pens (*n* = 10; five pens with barrows and five pens with gilts), and 3 pigs were housed in a pen (1.42 m × 3.86 m) with a concrete floor. Treatments were arranged based in a 2 × 2 factorial. The first factor was the antibiotic as growth promoter (AGP; 0 or 35 mg tylosin/kg diet; *n* = 20), and the second factor was the phytobiotics (PHY; 0 or 0.05%; *n* = 20). The experimental diets were formulated meeting the nutritional requirements suggested by NRC [20] and fed to pigs for 42 d based on 2 phases: grower 1 (d 1 to 21) and grower 2 (d 21 to 42). The composition of the basal diets is shown in Table 2.

The pigs (PIC 337× Camborough 22) used in the Exp. 1 and 2 were acquired from N.G. Purvis Farm (Ellerbe, NC, USA). Pigs from the same litter were randomly allotted to the treatments. The phytobiotics (By-O-reg^+^, Advanced Ag Products, Hudson, SD, USA) were composed of essential oils (carvacrol and thymol) from oregano (*Origanum vulgare*), soy hulls, aluminosilicate, limestone, dried *Saccharomyces cerevisiae* fermentation product, yeast, and soy oil in encapsulated forms. The feed additives were added to the basal diet by replacing corn. Pigs had free access to diets and water during Exp. 1 and 2. Feed intake and BW were recorded at the end of each phase to calculate ADG, ADFI, and G:F as indicators of growth performance. 

### 2.2. Sample Collection and Processing

In Exp. 1, at the end of the experimental period, blood samples were drawn from the jugular vein of all pigs into vacutainer tubes containing ethylenediamine tetraacetic acid (EDTA). Blood samples were centrifuged (1500× *g* at 4 °C for 10 min) in order to obtain plasma. The plasma was stored at −80 °C until analysis. All 40 pigs were euthanized by exsanguination after stunning by a captive bolt to collect intestinal tissue, mucosa, and digesta. Immediately after exsanguination, the gastrointestinal tract was removed to dissect the small intestine. Tissues (10 cm) from the duodenum (5 cm after the pyloric duodenal junction) and distal jejunum (20 cm before the ileum) were flushed with sterile saline solution and fixed in 10% formalin. Fixed tissues were used for Ki-67 staining, as an indicator of the crypt cell proliferation rate, and for assessments of intestinal morphology by measuring villus height, villus width, crypt depth, and the villus height to crypt depth ratio (VH:CD) [12]. 

Segments of the duodenum and distal jejunum from all pigs were used to collect mucosal scrapings. The mucosal samples were frozen in liquid nitrogen and stored at −80 °C immediately after collection for further processing. Duodenal and jejunal mucosa samples (0.5 g) were suspended in 1 mL of phosphate-buffered saline (PBS) and homogenized on ice using a tissue homogenizer (Tissuemiser; Thermo Fisher Scientific Inc., Waltham, MA, USA). After centrifugation at 10,000× *g* at 4 °C for 15 min. The supernatant was collected and divided into six aliquots and stored at −80 °C until analysis to measure tumor necrosis factor-alpha (TNF-α) as an indicator of the local inflammatory response, protein carbonyl (PC) as an indicator of local oxidative stress status, and immunoglobulin G (IgG) and immunoglobulin A (IgG) as indicators of local humoral immune status. Tissues from the distal jejunum were collected to measure tight junction proteins (claudin-1, occludin, and zonula occludens-1 [ZO-1]) as indicators of intestinal barrier function. Ileal digesta was collected into 50 mL containers and then frozen at −20 °C until being freeze-dried (24Dx48, Virtis, Gardiner, NY, USA). Dried digesta and feed samples were ground for analysis of the apparent ileal digestibility (AID) of dry matter (DM) gross energy (GE), ether extract (EE), and crude protein (CP).

In Exp. 2, at the end of the experimental period, 40 pigs representing the median BW of each pen (5 barrows and 5 gilts per treatment) were selected to collect blood samples from the jugular vein into vacutainer tubes containing ethylenediamine tetraacetic acid (EDTA). Plasma was obtained after centrifugation (1500× *g* at 4 °C for 10 min) and stored at −80 °C until analysis. Blood plasma samples were used to measure the concentration of TNF-α, PC, and IgG.

### 2.3. Immune and Oxidative Stress Status

The concentrations of protein, TNF-α, IgA, IgG, and PC in the plasma and mucosa of duodenum and jejunum were measured by colorimetric methods using a microplate reader (Synergy HT, Biotek Inc. Winooski, VT, USA) and the Gen 5 data analysis software (Biotek). The concentration of protein was measured using a BCA protein assay (23225; Thermo Fisher Scientific, Waltham, MA, USA) following the instructions of the manufacturer. The concentration of protein was used to normalize the concentration of TNF-α, IgA, IgG, and PC [21]. 

The concentration of TNF-α was measured using an ELISA kit (PTA00; R&D System, Minneapolis, MN, USA) following the procedures of the manufacturer [22]. The standard had a working range of 0 to 1500 pg/mL with a detection limit for TNF-α at 5 pg/mL. Absorbance was read at 450 nm and 550 nm, and the concentrations of TNF-α in mucosa and plasma were expressed as pg/mg and pg/mL protein, respectively. The concentration of IgA in mucosal samples was measured using the kit ELISA Pig IgA (E101-102, Bethyl Laboratories Inc, Montgomery, TX, USA) [9]. The concentration of IgG in plasma and mucosal samples was measured using the kit ELISA Pig IgG (E101-104, Bethyl Laboratories) as described by [23]. Absorbance was read at 450 nm, and the concentrations of IgA and IgG in mucosa and plasma were expressed as µg/mg of protein and mg/mL, respectively. 

The concentration of PC was measured using an ELISA kit (STA 310; Cell Biolabs, San Diego, CA, USA) [24]. Before the assay, plasma and mucosal samples were diluted in PBS to reach the protein concentration at 10 μg/mL. The absorbance was measured at 450 nm. The standard had a working range of 0 to 7.5 nmol/mg protein, and the concentrations of PC in mucosa and plasma were expressed as nmol/mg protein.

### 2.4. Tight Junction Proteins

Three samples of jejunum in each treatment were used to evaluate the expression of tight junction proteins as previously described by Kim et al. [25]. Within each treatment, the samples were selected based on the sex (two barrows and two gilts) and the median BW blocks. Briefly, jejunal samples (500 mg) were added to extraction buffer containing 0.5 mL RIPA lysis and 5 µL protease inhibitor mixture and homogenized at 4 °C. The supernatant was collected after centrifugation of the homogenized tissue at 10,000× *g* at 4 °C for 10 min. The concentration of protein in the supernatant was measured using a BCA protein assay (23225; Thermo Fisher Scientific Inc.) and was adjusted to 2 µg/µL. The samples (2 µg/µL protein) were denatured in the water bath for 5 min at 100 °C. The denatured samples were added in each well for SDS-PAGE. The gel was moved on a polyvinylidene difluoride (PVDF) membrane, and the target proteins were transferred to the membrane. Proteins were transferred at 90 mV for 1 h. Then, it was blocked in 5% skim milk, and incubated overnight at 4 °C with primary antibodies against β-actin (42 kDa), claudin-1 (22.7 kDa), occluding (59.1 kDa), and ZO-1 (220 kDa). Subsequently, the membrane was washed with wash solution and incubated for 1 h at room temperature with horseradish-conjugated secondary antibodies. A DAB substrate kit (34002; Pierce, Rockford, IL, USA) was used to develop the immunoblot. The LI-COR imaging system and the Image Studio Lite software (LI-COR Biosciences, Lincoln, NE, USA) was used to measure the density of bands of proteins. The protein expression was reported as band intensity (% of control) normalized with β-actin.

### 2.5. Intestinal Morphology and Crypt Cell Proliferation

A total of 40 samples of duodenum and 40 samples of jejunum fixed in 10% formalin were embedded in paraffin (two sections per cassette) after dehydration for immunohistochemistry assay. Briefly, the retrieval of the epitope was performed in a pressure cooker (Dako, Carpinteria, CA, USA) at using 10 mM citrate buffer with pH 6.0. Endogenous peroxidase was extinguished with 3% hydrogen peroxide. A protein block reagent (Dako) was used to block the sections. A primary monoclonal antibody of Ki-67 (Dako) was diluted (1:500) before be used. A secondary antibody was diluted (1:2) and added by using Vector ImmPRESS anti-mouse polymer reagent (Vector Laboratories, Burlingame, CA, USA). Diaminobenzidine (DAB) (Vector Laboratories) was used as the chromogen. 

Pictures of villi and their respective crypts were taken on each slide at a magnification of 40× using a microscope Olympus CX31 with a camera Infinity 2-2 digital CCD (Lumenera Corporation, Ottawa, Canada) following Jang et al. [26]. Lengths of 15 intact well-shaped villi and their respective crypts were measured in each slide to evaluate the villus height, villus width, and crypt depth. Pictures of 15 well-shaped crypts were taken per slide at a magnification of 100× to calculate the Ki-67^+^ cells using the Image JS software [27]. The ratio of Ki-67^+^ cells to total cells in the crypt (%) was used as an indicator of the crypt cell proliferation rate. All analyses of the intestinal morphology and immunohistochemistry were executed by the same trained person. 

### 2.6. Apparent Ileal Digestibility

Dried digesta and feeds were used to measure the titanium dioxide, GE, EE, and CP. Gross energy was measured using a calorimeter bomb (Parr 6200–Parr instrument company, Moline, IL, USA). The ether extract was measured following AOAC [28] (Method 920.39). Nitrogen was measured using TruSpec N Nitrogen Determinator (LECO Corp., St. Joseph, MI, USA) to calculate CP (Method 992.15; AOAC [28]). 

The AID of the nutrients was calculated using the following equation [29]:

AID = {1 − [(TiO_2feed_/TiO_2digesta_) × (Nutrient_digesta_/Nutrient_feed_)]} × 100 (1)

where TiO_2feed_ and TiO_2digesta_ were the concentrations of titanium dioxide in the diet and the digesta; Nutrient_digesta_ and Nutrient_feed_ were the concentrations of nutrients in the digesta and the diet.

### 2.7. Statistical Analysis

Data were analyzed based on a randomized complete block design using the Mixed procedure of SAS 9.4 Software (Cary, NC, USA). In Exp. 1 and 2, the experimental unit was a pen. The main effects were the factors (AGP and PHY) and their interaction. Factors and interactions were considered fixed effects, whereas sexes were considered random effects. When an interaction between the factors was significant or tended to be significant, a pairwise comparison was made using the PDIFF option in SAS. Statistical differences were considered significant with *p* < 0.05, whereas 0.05 ≤ *p* < 0.10 was used as the criteria for tendencies.

## 3. Results

### 3.1. Growth Performance

In Exp. 1, the BW of nursery pigs was not affected by the AGP or PHY factors (Table 3). The ADG of nursery pigs was not affected by the AGP or PHY factors during the experimental periods. However, during phase 1, there was a tendency for interaction (*p* = 0.078), where pigs fed an AGP diet tended to have greater ADG compared with pigs fed a diet without AGP or PHY. During phase 2, there was an interaction (*p* < 0.05) where pigs fed an AGP diet had greater ADG compared with pigs fed diet without AGP or PHY. During the overall period, there was an interaction (*p* < 0.05) where pigs fed an AGP diet had greater ADG compared with pigs fed diet without AGP or PHY and pigs fed a diet with AGP combined with PHY. The ADFI of nursery pigs was not affected by the AGP or PHY factors during nursery 1 and 2, whereas during the overall period, there was a tendency for interaction (*p* = 0.091) where pigs fed the AGP diet tended to have greater ADFI compared with pigs fed a diet without AGP or PHY. The G:F of nursery pigs was not affected by the AGP or PHY factors during phase 1. During phase 2, there was a tendency for interaction (*p* = 0.096) where pigs fed an AGP diet tended to have greater G:F compared with pigs fed a diet with AGP combined with PHY. During the overall period, there was an interaction (*p* < 0.05) where pigs fed an AGP diet had greater (*p* < 0.05) G:F compared with pigs fed a diet without AGP or PHY and pigs fed a diet with AGP combined with PHY.

In Exp. 2, the BW of growing pigs was not affected by the AGP or PHY factors (Table 4) during 21 d of the experiment. On d 42, there was an interaction (*p* < 0.05) where pigs fed solely AGP or PHY diets had greater BW compared with pigs fed a diet with no AGP and PHY inclusion and pigs fed a diet with AGP combined with PHY. The ADG and ADFI of growing pigs were not affected by the AGP or PHY factors during grower 1. During grower 2 and the overall period, there was an interaction (*p* < 0.05) where pigs fed solely AGP or PHY diets had greater ADG compared with pigs fed a diet without AGP or PHY and pigs fed a diet with AGP combined with PHY. During grower 2, there was an interaction (*p* < 0.05) where pigs fed a diet with AGP combined with PHY had lower ADFI compared with pigs fed diets without AGP or PHY and diets with AGP or PHY. During the overall period, there was an interaction (*p* < 0.05) where pigs fed solely AGP or PHY diets had greater ADFI compared with pigs fed a diet with AGP combined with PHY. During grower 1, AGP diets increased (*p* < 0.05) the G:F, whereas PHY diets did not affect the G:F growing pigs. During grower 2, PHY diets increased (*p* < 0.05) the G:F, whereas AGP diets did not affect the G:F growing pigs. During the overall period, AGP and PHY diets increased (*p* < 0.05) the G:F of growing pigs.

### 3.2. Immune and Oxidative Stress Status

In Exp. 1, the concentration of TNF-α in the duodenal mucosa and plasma of nursery pigs was not affected by the AGP or PHY factors (Table 5). The PHY diets decreased (*p* < 0.05) the concentration of TNF-α in jejunal mucosa in comparison to the diets without PHY. The AGP diet tended to decrease (*p* = 0.067) the concentration of IgG in the duodenal mucosa, whereas the PHY diet decreased (*p* < 0.05) it in jejunal mucosa and tended to decrease (*p* = 0.064) it in plasma. The concentration of IgA in the duodenal mucosa and plasma of the nursery pigs was not affected by the factors. The AGP diet tended to decrease (*p* = 0.063) the concentration of IgA in the duodenal mucosa. The concentration of PC in the duodenal and jejunal mucosa of nursery pigs was not affected by the factors. The PHY diet decreased (*p* < 0.05) the concentration of PC in the plasma of nursery pigs.

In Exp. 2, the concentration of TNF-α in the plasma of grower pigs was not affected by the AGP or PHY factors (Table 6). The PHY diet decreased (*p* < 0.05) the concentration of IgG and PC in the plasma of grower pigs regardless of the AGP supplementation.

### 3.3. Tight Junction Proteins

**Figure 1:**
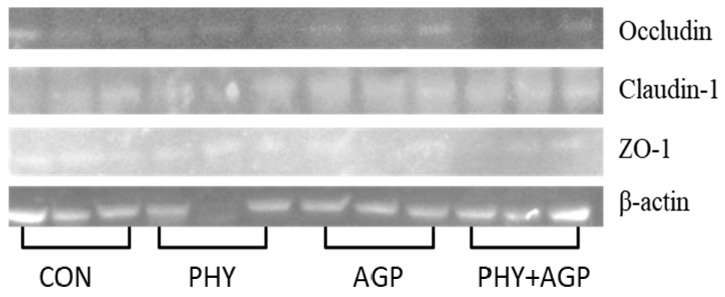
Bands of tight junction proteins in the jejunal tissue of weaning pigs fed a diet supplemented with phytobiotics and antibiotics as growth promoters (Exp. 1). AGP and PHY factors and their interaction (*n* = 3 per treatment; *n* = 12 total).

In Exp. 1, the expression of Occludin tended to increase (*p* < 0.053) in the jejunum of nursery pigs fed PHY diets (Table 7 and Figure 1), whereas it was reduced in the jejunum of nursery pigs fed a diet with AGP combined with PHY. The expressions of Claudin-1 and ZO-1 were not affected by the factors.

### 3.4. Intestinal Morphology and Crypt Cell Proliferation

In Exp. 1, the duodenal morphology of nursery pigs was not affected by the AGP or PHY factors (Table 8). The PHY diets increased (*p* < 0.05) the villus height in the jejunum of nursery pigs. There was an interaction (*p* < 0.05) where the AGP diet increased the enterocyte proliferation in crypts of jejunum compared with the diet without PHY, whereas there was no effect when the diet contained PHY. The villus width, crypt depth, and VH:CD ratio were not affected by AGP or PHY factors.

### 3.5. Apparent Ileal Digestibility

In Exp. 1, the AGP tended to increase the AID of DM (*p* = 0.099) and CP (*p* = 0.065) (Table 9). The AID of GE tended to be greater (*p* = 0.090) in pigs fed a PHY diet compared with those fed the diet without AGP and PHY. The AID of EE was not affected by the factors.

## 4. Discussion

The use of AGP has been restricted or banned in several countries around the world due to the concern over microbial resistance [4,6,7,8]. Alternative feed additives to replace or reduce the use of antibiotics in feeds should improve the growth of animals by improving their health status without residual or microbial resistance concerns [4,30]. This study showed that phytobiotics, similarly to AGP, increased the daily gain, feed intake, and feed efficiency of pigs during the growing phase when supplemented solely, whereas it suppressed the AGP effects during the nursery and growing phases when they were supplemented in conjunction. The mode of action of phytobiotics depends on the composition of the active ingredients in the product. Phenolic compounds are the main bioactive compounds in phytobiotics, and their composition and concentration vary according to the plant, parts of the plant, geographical origin, harvesting season, environmental factors, storage conditions, and processing techniques [18,31]. The phytobiotics used in the current study are based on oregano oils that are mainly composed of carvacrol, thymol, γ-terpinene, and *p*-cymene [18]. Carvacrol and thymol have been reported to exert antimicrobial, anti-inflammatory, and anti-oxidative properties that can enhance intestinal health and consequently improve growth performance [17,19,32]. According to Wang et al. [33], carvacrol and thymol increased the growth performance of nursery pigs by modulating the microbiota and improving intestinal barrier function. Furthermore, oregano essential oil fed to lactating sows has been reported to improve the offspring’s performance [19].

The enhanced growth performance observed in pigs fed phytobiotics in the current study could be associated with the reduction in the concentration of TNF-α in the jejunum and IgG in the jejunum and plasma of nursery and growing pigs. The jejunum accounts for critical functions in the immune system, in addition to the digestive role [4,21]. One proposed mechanism of phytobiotics affecting the immune system is through the modulation of intestinal microbiota, which in turn would reduce inflammatory response [17,34,35]. Additionally, Lima et al. [36] proposed that the anti-inflammatory properties of carvacrol are related to its ability to induce IL-10 production, consequently reducing the release of IL-1β and prostaglandin E_2_. The inhibition or reduction of pro-inflammatory cytokine production is imperative to enhance health and promote growth performance [4]. However, the improved growth performance was observed in pigs fed diets with solely phytobiotics in the growing phase. The use of AGP improved the growth performance, without affecting the inflammatory and oxidative stress status of pigs during the nursery and growing phases.

Production of anti-inflammatory and pro-inflammatory mediators, including cytokines and reactive oxygen species (ROS), occurs naturally in a homeostasis status. The overproduction of pro-inflammatory mediators may cause the exhaustion of the antioxidant mechanism, leading to the oxidation of cellular organic compounds including DNA, RNA, lipids, and proteins [37]. According to Gebicki [38], proteins are the main target for ROS, and their oxidation results in protein carbonyl through an irreversible and stable reaction [39]. Therefore, protein carbonyl is an important marker of oxidative stress status [39,40,41]. In this study, the addition of phytobiotics reduced the concentration of protein carbonyl in the plasma of pigs during the nursery and growing period. These results confirmed the antioxidant properties of oregano-based phytobiotics, as previously reported [32,35,42]. According to Zou et al. [32], oregano essential oil diminished the oxidative stress in IPEC-J2 cells by inducing the expression of superoxide dismutase (SOD1) and glutathione through the activation of nuclear-factor-erythroid-2-related factor-2 (Nrf2), which can be one of the anti-oxidant modes of action of oregano-based phytobiotics. Furthermore, according to Rodriguez-Garcia et al. [35] oregano essential oil can scavenge free radicals including ROS and, consequently, reduce the oxidative damage to organic molecules.

The oxidative stress compounds can damage cellular macromolecules, increasing senescence and apoptosis and consequently reducing villus and increasing cell proliferation [37,43,44]. In the current study, phytobiotics or AGP did not affect the expression of tight junction proteins in the jejunum of nursery pigs, although previous studies have reported that phytobiotics and AGP play a role in the expression of these proteins [45]. Interestingly, when AGP and phytobiotics were supplemented in combination, the expression of occludin was reduced, which may explain the results of growth performance and digestibility observed in the current study. It seems that the combinational addition of phytobiotics and AGP may have inhibited their individual effects. However, essential oils have been reported to have synergetic antimicrobial activity when used combined with antibiotics [46]. 

The antioxidant property of phytobiotics confers a protective effect on the intestinal barrier function. The reduced oxidative stress has been associated with enhanced intestinal morphology [4,43]. In this study, the phytobiotics increased the villus height in the jejunum of nursery pigs, without affecting the enterocyte proliferation in the jejunal crypt. These findings are in agreement with Namkung et al. [47] and Murugesan et al. [48], who indicate that phytobiotics supplementation to pig and poultry feed can improve the villus height. Interestingly, the AGP increased the enterocyte proliferation without affecting other parameters related to intestinal morphology. Zou et al. [45] reported that the essential oil of oregano enhanced the intestinal barrier function by increasing the villus height and the expression of tight junction proteins. The authors associated these results with the reduction in the expression of TNF-𝛼, IL-1𝛽, IL-6, MCP-1, and INF-γ. 

The reduced oxidative stress and inflammatory status may also have contributed to promoting the growth of pigs in this study through reduced metabolic cost for immune responses and to repairing damages in the intestinal epithelium. The changes in intestinal morphology may partially explain the trend to increase the AID of GE observed in pigs fed diets with phytobiotics. Pigs fed AGP showed a trend to increase the AID of dry matter and CP. A greater villus height is generally correlated with increased nutrient digestibility [49]. Furthermore, phytobiotics have also been shown to increase pancreatic enzyme production and activity and increase bile secretion, which would increase nutrient digestibility [50,51]. 

The use of encapsulation or coating is a fundamental process to stabilize certain feed additives reducing unpleasant odor and taste, as well as to slow-release the bioactive compound along the intestine [52,53,54,55]. The phytobiotics used in this study were encapsulated to stabilize the bioactive compounds and slow-release them along the intestine. As aforementioned, phytobiotics reduced inflammatory and oxidative stress in the jejunum, whereas no effect was observed in the duodenum. The lack of effects on intestinal health parameters in the duodenum observed in pigs fed phytobiotics indicates that the coating may have released the bioactive compounds mostly in the jejunum. Considering the digestive and immunologic functions, the jejunum is a key site to target the release of antibiotic and antioxidant compounds in phytobiotics. 

## 5. Conclusions

In conclusion, phytobiotics, similarly to antibiotics, improved the growth performance of pigs from the weaning to growing phases by reducing inflammatory reaction, oxidative stress, and the humoral immune response, and consequently, enhancing the jejunal morphology. However, the combinational use of phytobiotics with antibiotics suppressed their effects. 

## Figures and Tables

**Table 1:** Composition of basal diets (Exp. 1; as-fed basis).

Item	Nursery 1	Nursery 2
Ingredient, %		
Corn, yellow dent	38.5	47.6
Soybean meal, 48% CP	23.0	26.0
Whey permeate	21.0	13.0
Blood plasma (porcine)	5.0	2.0
Poultry meal	5.0	5.0
Fish meal	3.0	2.0
L-Lys HCl	0.38	0.35
DL-Met	0.20	0.15
L-Thr	0.12	0.10
Dicalcium phosphate	0.20	0.45
Limestone	0.78	0.75
Mineral premix ^1^	0.15	0.15
Vitamin premix ^2^	0.03	0.03
Sodium chloride	0.22	0.22
Zinc oxide	0.25	0.25
Poultry fat	2.20	2.00
Calculated composition		
ME, Mcal/kg	3.42	3.41
SID ^3^ Lys, %	1.50	1.35
SID Met + Cys, %	0.82	0.74
SID Thr, %	0.89	0.79
SID Trp, %	0.26	0.22
Ca, %	0.86	0.81
STTD ^4^ P, %	0.46	0.41
Analyzed composition		
Dry matter, %	92.95	92.47
Crude protein, %	22.22	22.26
Ca, %	0.85	0.76
P, %	0.64	0.65
NDF ^5^, %	7.15	6.77
ADF ^6^, %	2.63	3.66

^1^ The trace mineral premix supplied the following per kg of complete diet: 16.5 mg of Cu as CuSO_4_, 0.3 mg I as ethylenediamine dihydriodide, 165 mg of Fe as FeSO_4_, 40 mg of Mn as MnSO_4_, 0.3 mg of Se as Na_2_SeO_3_, and 165 mg of Zn as ZnO. ^2^ The vitamin premix supplied the following per kg of complete diet: 8433 IU of vitamin A, 1202 IU of vitamin D3 as activated animal sterol, 48 IU of vitamin E, 4.0 mg of vitamin K as menadione dimethylpyrimidinol bisulfate, 6.0 mg of riboflavin, 36.2 mg of niacin, 24.1 mg of d-pantothenic acid as calcium pantothenate, 1.8 mg of folic acid, 0.24 mg of d-biotin, 0.031 mg of vitamin B12. ^3^ SID: Standardized ileal digestibility. ^4^ STTD P, standardized total tract digestible phosphorus. ^5^ Neutral detergent fiber. ^6^ Acid detergent fiber.

**Table 2:** Composition of basal diets (Exp. 2; as-fed basis).

Item	Grower 1	Grower 2
Ingredient, %		
Corn, yellow dent	70.2	76.6
Soybean meal, 48% CP	25.0	20.0
L-Lys HCl	0.24	0.23
DL-Met	0.04	0.01
L-Thr	0.04	0.04
Mineral premix ^1^	0.15	0.15
Vitamin premix ^2^	0.03	0.03
Dicalcium phosphate	1.05	0.90
Limestone	0.85	0.80
Sodium chloride	0.20	0.20
Poultry fat	2.20	1.00
Calculated composition		
ME, Mcal/kg	3.40	3.36
SID ^3^ Lys, %	0.98	0.85
SID Cys + Met, %	0.55	0.48
SID Thr, %	0.59	0.52
SID Trp, %	0.18	0.16
Ca, %	0.66	0.59
STTD ^4^ P, %	0.31	0.27
Analyzed Composition		
Dry matter, %	91.53	91.01
Crude protein, %	16.28	14.94
Ether extract, %	4.60	3.42
Ca, %	0.67	0.71
P, %	0.53	0.51
NDF ^5^, %	7.30	6.94
ADF ^6^, %	3.21	3.16

^1^ The trace mineral premix supplied the following per kg of complete diet: 16.5 mg of Cu as CuSO_4_, 0.3 mg I as ethylenediamine dihydriodide, 165 mg of Fe as FeSO_4_, 40 mg of Mn as MnSO_4_, 0.3 mg of Se as Na_2_SeO_3_, and 165 mg of Zn as ZnO. ^2^ The vitamin premix supplied the following per kg of complete diet: 8433 IU of vitamin A, 1202 IU of vitamin D3 as activated animal sterol, 48 IU of vitamin E, 4.0 mg of vitamin K as menadione dimethylpyrimidinol bisulfate, 6.0 mg of riboflavin, 36.2 mg of niacin, 24.1 mg of d-pantothenic acid as calcium pantothenate, 1.8 mg of folic acid, 0.24 mg of d-biotin, 0.031 mg of vitamin B12. ^3^ SID: Standardized ileal digestibility. ^4^ STTD P, standardized total tract digestible phosphorus. ^5^ Neutral detergent fiber. ^6^ Acid detergent fiber.

**Table 3:** Growth performance of nursery pigs fed diets supplemented with phytobiotics and antibiotics as growth promoters (Exp. 1).

Antibiotic (AGP)	−		+		SEM	*p* Value ^1^		
Phytobiotic (PHY)	−	+	−	+		AGP	PHY	AGP × PHY
BW, kg								
Initial	6.4	6.4	6.3	6.4	0.3	0.461	0.650	0.866
d 7	6.7	6.9	7.0	6.7	0.4	0.723	0.664	0.270
d 21	12.6	13.4	13.5	12.8	0.6	0.740	0.904	0.106
ADG, g/d								
Phase 1 (d 1 to 7)	44 ^B^	59 ^AB^	96 ^A^	51 ^AB^	17	0.184	0.367	0.078
Phase 2 (d 7 to 21)	424 ^b^	475 ^ab^	488 ^a^	430 ^ab^	28	0.709	0.892	0.047
Overall	292 ^b^	334 ^ab^	368 ^a^	304 ^b^	20	0.212	0.545	0.007
ADFI, g/d								
Phase 1 (d 1 to 7)	121	127	156	122	15	0.295	0.347	0.181
Phase 2 (d 7 to 21)	530	578	574	551	30	0.765	0.654	0.203
Overall	391 ^B^	421 ^AB^	446 ^A^	408 ^AB^	22	0.280	0.831	0.091
G:F								
Phase 1 (d 1 to 7)	0.34	0.46	0.51	0.41	0.08	0.410	0.900	0.163
Phase 2 (d 7 to 21)	0.80 ^AB^	0.82 ^AB^	0.85 ^A^	0.78 ^B^	0.03	0.846	0.339	0.096
Overall	0.75 ^b^	0.79 ^ab^	0.82 ^a^	0.75 ^b^	0.03	0.627	0.526	0.026

^1^ AGP and PHY factors and their interaction (*n* = 10 per treatment; *n* = 40 total). ^a,b^ Means within a row with different superscripts differ (*p* < 0.05). ^A,B^ Means within a row with different superscripts show a tendency to differ (0.05 ≤ *p* < 0.10).

**Table 4:** Growth performance of growing pigs fed diets supplemented with phytobiotics and antibiotics as growth promoters (Exp. 2).

Antibiotic (AGP)	−		+		SEM	*p* Value ^1^		
Phytobiotic (PHY)	−	+	−	+		AGP	PHY	AGP × PHY
BW, kg								
Initial	27.9	28.0	27.9	28.0	2.3	0.972	0.721	0.957
d 21	52.3	52.5	53.0	52.5	3.2	0.425	0.802	0.395
d 42	72.0 ^b^	74.5 ^a^	74.6 ^a^	72.7 ^ab^	4.23	0.658	0.721	0.009
ADG, kg/d								
Grower 1 (d 1 to 21)	1.16	1.17	1.20	1.17	0.04	0.222	0.456	0.220
Grower 2 (d 21 to 42)	1.10 ^b^	1.22 ^a^	1.20 ^a^	1.12 ^b^	0.07	0.980	0.450	0.002
Overall	1.13 ^b^	1.19 ^a^	1.20 ^a^	1.14 ^b^	0.06	0.609	0.783	0.003
ADFI, g/d								
Grower 1 (d 1 to 21)	2.22	2.27	2.26	2.20	0.13	0.620	0.817	0.156
Grower 2 (d 21 to 42)	2.75 ^a^	2.80 ^a^	2.81 ^a^	2.58 ^b^	0.21	0.164	0.107	0.014
Overall	2.49 ^ab^	2.53 ^a^	2.54 ^a^	2.39 ^b^	0.16	0.253	0.237	0.023
G:F								
Grower 1 (d 1 to 21)	0.52	0.52	0.53	0.54	0.01	0.025	0.695	0.454
Grower 2 (d 21 to 42)	0.40 ^B^	0.44 ^A^	0.43 ^A^	0.44 ^A^	0.01	0.110	0.005	0.060
Overall	0.46	0.47	0.47	0.48	0.01	0.013	0.022	0.273

^1^ AGP and PHY factors and their interaction (*n* = 10 per treatment; *n* = 40 total). ^a,b^ Means within a row with different superscripts differ (*p* < 0.05). ^A,B^ Means within a row with different superscripts show a tendency to differ (0.05 ≤ *p* < 0.10).

**Table 5:** Inflammatory and oxidative stress status in the jejunum of nursery pigs fed diets supplemented with phytobiotics and antibiotics as growth promoters (Exp. 1).

Antibiotic (AGP)	−		+		SEM	*p* Value ^1^		
Phytobiotic (PHY)	−	+	−	+		AGP	PHY	AGP × PHY
Duodenum								
TNF-α ^2^, pg/mg protein	3.79	3.91	4.07	4.06	0.33	0.481	0.863	0.842
IgG ^3^, μg/mg protein	2.10	1.58	1.45	1.41	0.24	0.067	0.202	0.268
IgA ^4^, μg/mg protein	1.68	1.55	1.38	1.43	0.16	0.161	0.767	0.563
PC ^5^, nmol/mg protein	7.01	6.56	7.10	7.15	0.73	0.598	0.762	0.698
Jejunum								
TNF-α, pg/mg protein	4.62	3.90	4.19	3.74	0.30	0.253	0.031	0.606
IgG, μg/mg protein	1.46	1.09	1.37	1.29	0.11	0.586	0.037	0.150
IgA, μg/mg protein	1.83	1.36	1.22	1.06	0.27	0.063	0.190	0.518
PC, nmol/mg protein	8.69	7.11	7.65	7.37	0.68	0.541	0.161	0.321
Plasma								
TNF-α, pg/mL	63.7	62.5	64.0	58.3	10	0.842	0.732	0.822
IgG, mg/mL	2.36	2.15	2.31	1.85	0.21	0.312	0.064	0.473
IgA, mg/mL	0.25	0.23	0.27	0.24	0.04	0.601	0.419	0.949
PC, nmol/mg protein	4.34	3.02	4.06	2.77	0.45	0.489	0.002	0.973

^1^ AGP and PHY factors and their interaction (*n* = 10 per treatment; *n* = 40 total). ^2^ Tumor necrosis factor alpha. ^3^ Immunoglobulin G. ^4^ Immunoglobulin A. ^5^ Protein carbonyl.

**Table 6:** Inflammatory response, humoral immunity, and oxidative stress in the plasma of growing pigs fed diets supplemented with phytobiotics and antibiotics as growth promoters (Exp. 2).

Antibiotic (AGP)	−		+		SEM	*p* Value ^1^		
Phytobiotic (PHY)	−	+	−	+		AGP	PHY	AGP × PHY
TNF-α ^2^, pg/mL	72.33	81.89	75.45	75.67	4.49	0.659	0.171	0.190
IgG ^3^, mg/mL	10.42	9.33	10.38	8.18	0.61	0.341	0.011	0.370
PC ^4^, nmol/mg protein	5.21	3.88	5.45	4.26	0.32	0.341	<0.001	0.827

^1^ AGP and PHY factors and their interaction (*n* = 10 per treatment; *n* = 40 total). ^2^ Tumor necrosis factor alpha. ^3^ Immunoglobulin G. ^4^ Protein carbonyl.

**Table 7:** Tight junction proteins in jejunal tissue of nursery pigs fed diets supplemented with phytobiotics and antibiotics as growth promoters (Exp. 1).

Antibiotic (AGP)	−		+		SEM	*p* Value ^1^		
Phytobiotic (PHY)	−	+	−	+		AGP	PHY	AGP × PHY
Item, band intensity (% of control) normalized with β-actin								
Occludin	0.161 ^b^	0.185 ^b^	0.212 ^b^	0.070 ^a^	0.036	0.265	0.053	0.010
Claudin-1	0.233	0.294	0.270	0.232	0.037	0.717	0.740	0.165
ZO-1 ^2^	0.249	0.306	0.243	0.154	0.089	0.354	0.840	0.385

^1^ AGP and PHY factors and their interaction (*n* = 10 per treatment; *n* = 40 total). ^2^ Zonula occludens-1. ^a,b^ Means within a row with different superscripts differ (*p* < 0.05).

**Table 8:** Intestinal morphology and crypt cell proliferation in nursery pigs fed diets supplemented with phytobiotics and antibiotics as growth promoters (Exp. 1).

Antibiotic (AGP)	−		+		SEM	*p* Value ^1^		
Phytobiotic (PHY)	−	+	−	+		AGP	PHY	AGP × PHY
Duodenum								
Villus height, μm	465	460	473	462	16	0.790	0.663	0.849
Villus width, μm	145	146	142	148	4.9	0.902	0.515	0.593
Crypt depth, μm	257	249	252	251	8.2	0.910	0.573	0.655
VH:CD ^2^	1.88	1.87	1.88	1.87	0.10	0.752	0.836	0.728
Jejunum								
Villus height, μm	437	466	448	475	15	0.422	0.037	0.940
Villus width, μm	255	249	250	256	13	0.926	0.996	0.575
Crypt depth, μm	143	145	142	142	4.1	0.596	0.879	0.853
VH:CD	3.09	3.25	3.21	3.44	0.15	0.311	0.191	0.822
Ki-67 ^+3^, %	37 ^b^	44 ^ab^	48 ^a^	40 ^ab^	3.7	0.517	0.887	0.046

^1^ AGP and PHY factors and their interaction (*n* = 10 per treatment; *n* = 40 total). ^2^ Villus height to crypt depth ratio. ^3^ Enterocyte proliferation rate in the crypt. ^a,b^ Means within a row with different superscripts differ (*p* < 0.05).

**Table 9:** Apparent ileal digestibility of nutrients in nursery pigs fed diets supplemented with phytobiotics and antibiotics as growth promoters (Exp. 1).

Antibiotic (AGP)	−		+		SEM	*p* Value ^1^		
Phytobiotic (PHY)	−	+	−	+		AGP	PHY	AGP × PHY
Item, %								
Dry matter	63.5	65.2	65.8	66.9	1.2	0.099	0.229	0.805
Ether extract	66.7	68.5	67.6	68.1	1.8	0.817	0.333	0.595
Crude protein	71.4	72.4	75.9	73.6	1.8	0.065	0.679	0.274
Gross energy	65.5 ^A^	69.2 ^B^	68.4 ^AB^	68.0 ^AB^	1.2	0.452	0.166	0.090

^1^ AGP and PHY factors and their interaction (*n* = 10 per treatment; *n* = 40 total). ^A,B^ Means within a row with different superscripts show a tendency to differ (0.05 ≤ *p* < 0.10).

## Data Availability

Not applicable.
